# Repair of popliteomeniscal fascicles tear using a posterior transseptal portal fixes hypermobile lateral meniscus

**DOI:** 10.1186/s40634-021-00412-4

**Published:** 2021-10-21

**Authors:** Sohrab Keyhani, Mohammad Movahedinia, Mehran Soleymanha, Rene Verdonk, Morteza Kazemi, Mohamad Qoreishy

**Affiliations:** 1 Bone Joint and Related Tissues Research Center, Akhtar Orthopedic Hospital, Shahid Beheshti University of Medical Sciences, Sharifi manesh street, Shariati street, Tehran, Iran; 2 Guilan University of Medical Sciences, Rasht, Iran; 3 Department of Orthopedics and Traumatology, Gent University, Ghent, Belgium

**Keywords:** Hypermobile lateral meniscus, Lateral meniscus, Popliteomeniscal fascicles, Posterolateral portal, Posteromedial portal

## Abstract

**Purpose:**

This study investigates the effects of the all-inside repair of posterosuperior popliteomeniscal fascicle (PMF) on lateral meniscus stabilization using a posterior arthroscopic approach.

**Methods:**

Between 2015 and 2018, 17 patients with hypermobile lateral meniscus (HLM) underwent posterior knee arthroscopy for PMF repair. The all-inside repair was performed through posteromedial transseptal and posterolateral portals using a suture hook technique. Patients were clinically assessed based on IKDC and Lysholm scores.

**Results:**

Both IKDC and Lysholm scores improved significantly after an average follow-up of 3.5 years (*P* < 0.001). No patients underwent reoperation, and no complications associated with posterior knee arthroscopy were reported.

**Conclusion:**

The all-inside suture hook technique using posterolateral and posteromedial transseptal portals fixes HLM with excellent IKDC and Lysholm scores.

**Level of evidence:**

Level IV.

## Background

Hypermobile lateral meniscus (HLM) occurs following the disruption of the popliteomeniscal fascicles (PMF), the most frequent cause, near the popliteal tendon [1, 2]. In the case of HLM, the posterior portion of the lateral meniscus shows forward abnormal translation with knee flexion and backward translation with knee extension, opposing its physiological motion [3].

Patients do not typically present a specific history of trauma [4, 5]. A displaced lateral meniscus can be spontaneously reduced without any obvious tears observed via magnetic resonance imaging (MRI) [6–8]. Patients typically complain of knee locking or pain in the absence of a torn or discoid meniscus [9]. LaPrade and Konowalchuk described a clinical test that can reproduce the locking mechanism based on a figure-of-four position related to flexion, varus, and external rotation [10]. However, the gold standard for diagnosis is arthroscopic visualization of the popliteomeniscal ligaments at the popliteal hiatus, combined with an evaluation of lateral meniscal movements [11].

Surgical HLM treatment is generally recommended only for patients whose symptoms have not improved with conservative treatment [12, 13]. Different treatments have been proposed for the recurrent subluxation of the lateral meniscus, including arthroscopic subtotal meniscectomy or meniscus repair and thermal shrinkage of the posterolateral capsule [4, 10, 14, 15]. Osteoarthritic changes and joint locking have been reported in such treatments [12]. Current studies recommend the direct repair of the popliteomeniscal junction using different arthroscopic repair techniques [16–18].

The arthroscopic repair of the posterior horn of the lateral meniscus can be challenging because it is difficult to make an arthroscopic assessment in the anatomically narrow posterolateral compartment and because this area is anatomically complex [19, 20]. Although clinical studies have evaluated the role of arthroscopy in the treatment of recurrent lateral meniscus subluxation, the optimal techniques for HLM remains unknown [9].

An ideal treatment involves the anatomical and functional fixation of the lateral meniscus without interfering with the normal movement of the knee [21]. The purpose of this study is to evaluate the role of posterior knee arthroscopy in the all-inside repair of HLM using the suture hook technique. We hypothesized that disrupted PMF could be repaired by all-inside vertical mattress sutures using the posterior transseptal portal, thereby fixing HLM.

## Materials and methods

The indication of the surgery was defined by the experience of knee pain, locking, or snapping despite undergoing 6 months of conservative treatment. Hypermobility was confirmed when the excessive translation of the lateral meniscus was detected during arthroscopic probing. All patients presented a non-locked lateral meniscus with a popliteomeniscal tear in the posterior third portion (zone F0 according to cooper classification) [22]. Patients with open physis, discoid, or degenerative lateral meniscus were excluded. Clinical follow-up less than 6-month, and unstable knee were the other exclusion criteria.

Approval was acquired from the Institutional Research Ethics Committee. Written informed consent was obtained from all patients before the study began. Preoperative International Knee Documentation Committee (IKDC) and Lysholm scores were obtained before treatment and again at the final follow-up.

### Surgical technique

**Fig. 1:**
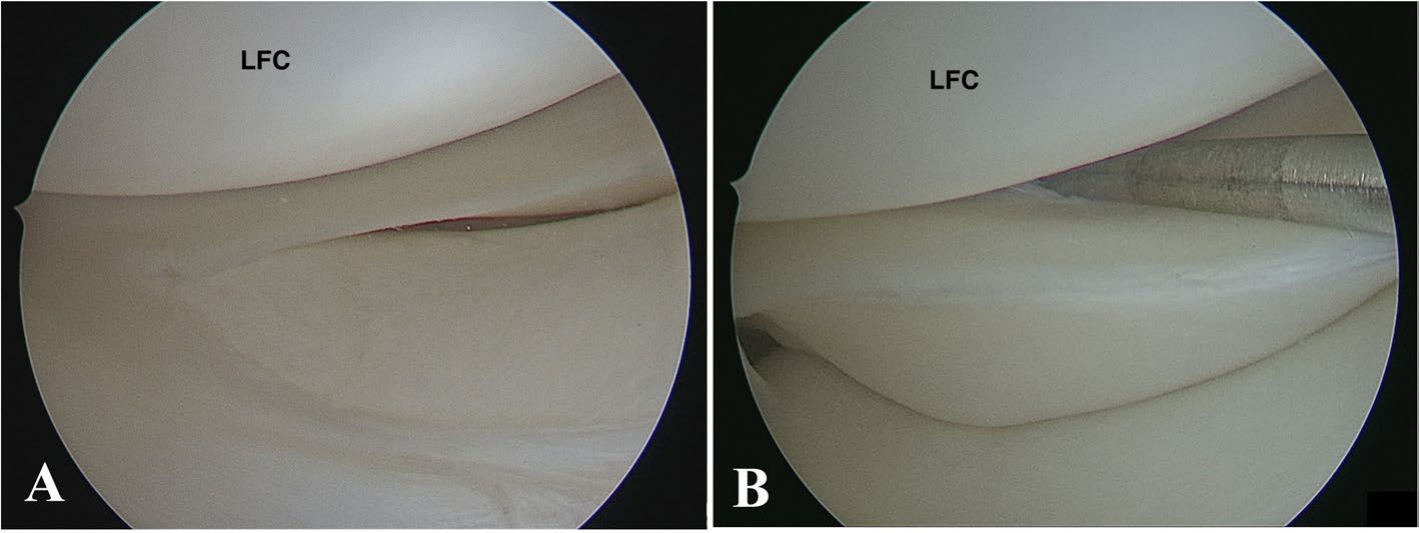
Right knee arthroscopy: **A** Anterolateral portal view shows normal lateral meniscus; **B** Abnormal lateral meniscal movement by probing

**Fig. 2:**
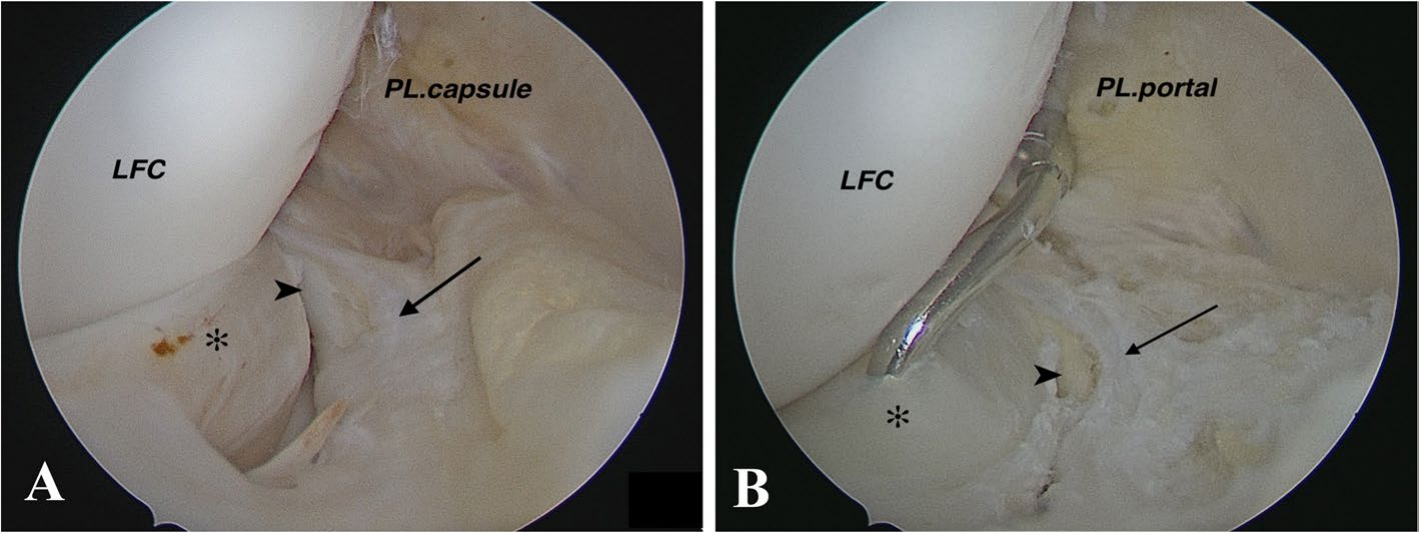
Right knee arthroscopy: **A** Posteromedial transseptal view with a 30-degree lens that shows popliteomeniscal fascicle tear (**B**) repair by using suture hook technique from posterolateral portal. Asterisk: Lateral meniscus; Arrow: Posterosuperior popliteomeniscal tear; Arrow head: Popliteus tendon

Surgical procedures were performed with patients in the supine position and under general anesthesia. The knee was flexed 90 degrees by draping the leg over the edge of the operating table. The meniscus movement was evaluated by a probe from an anteromedial portal in a figure-of-four position. If the meniscus was unstable during probing (Fig. 1) and the trans-notch visualization confirmed the presence of a meniscal tear in the posterior zone (zone F0), fixation surgery was performed. All procedures were carried out with a 30-degree lens. Directly following Gillquist’s view, the posteromedial portal was created in a soft spot area. The posteromedial and posterolateral portals were then created according to Keyhani et al.’s description (Fig. 2A) [23]. The instruments were introduced from the posterolateral portal without using a cannula.

**Fig. 3:**
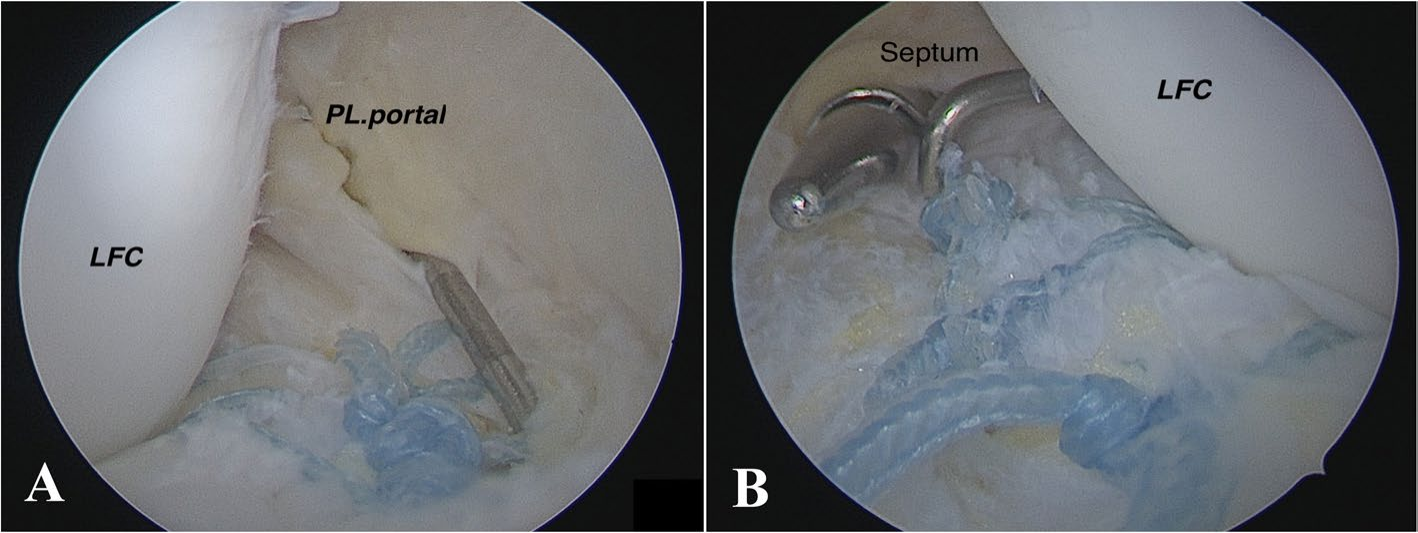
Repaired lateral meniscus: **A** Posteromedial transseptal and **B** posterolateral views

The meniscal border and adjacent synovium were abraded to enhance the chance of healing. The all-inside method was performed using a loaded suture hook (Lasso ConMed-Linvatec, Utica, NY, USA) with a No. 1 PDS (Ethicon; Somerville, NJ, USA) according to Keyhani et al.’s description. (Fig. 2B) [24]. Then PDS was replaced with a fiber wire (no. 2) (fiber wire; Arthrex, Naples, Fla). When simultaneous penetration was impossible, a shuttle relay system was used to lift the peripheral sagging fragment. A sliding SMC knot was applied to the meniscus with the help of a knot pusher, followed by three simple knots over the sliding knot in a different direction. This vertical mattress suture was repeated every 5–10 mm as needed. The suture hook was passed from the superficial part of the lateral meniscus to the soft tissue around the popliteus tendon so that the last suture could be placed in the most lateral part of the meniscus and at the point nearest to the popliteus tendon (Fig. 3).

After meniscal fixation, the adequate stability of the meniscus was confirmed with the probe in a figure-of-four position from the anterior. Finally, a 7-mm tunnel was created in the notch area using a femoral ACL reamer to further enhance healing potential and create a condition similar to ACL reconstruction [25].

A limited-motion brace was applied after 4 weeks of using the full-extension splint. The affected knee joint was allowed a gradual range of motion to achieve at least 90 degrees of flexion over 8 weeks. Patients were encouraged to walk with crutches 2 weeks after the surgery with partial weight-bearing. Full weight-bearing was allowed 12 weeks after the operation. A return to the pre-injury status and normal sports activities was allowed after 6 months of rehabilitation.

### Statistical analysis

SPSS software version 16 (IBM; Armonk, NY, USA) was used. The paired t-test was employed to compare the pre-and postoperative parametric variables (IKDC and Lysholm scores). *P* < 0.001 was considered as a significant threshold.

## Results

This study includes 17 patients—10 men and seven females—diagnosed with HLM. They were operated on between 2015 and 2018, with an average follow-up of 3.5 years (ranging from 3 to 5 years). The mean age at the time of operation was 34 ± 6 years (range 18–42 years). A PMF tear was evident from MRI for three patients. For all other patients, the diagnosis was confirmed by surgery, as there was no evidence of PMF tearing from their MRI scans.

**Fig. 4:**
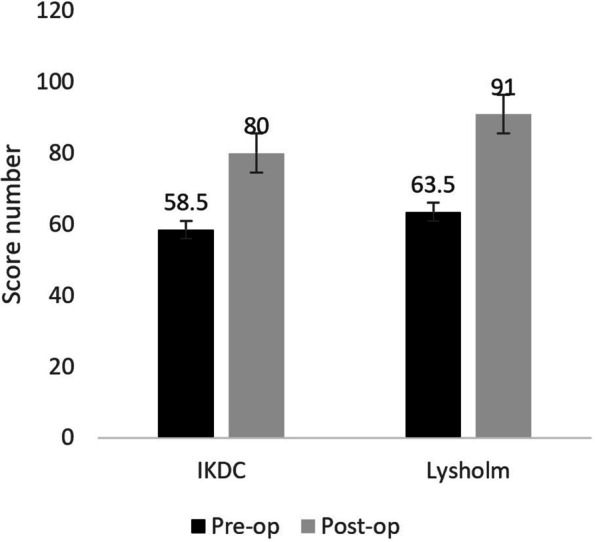
Pre- and postoperative clinical scores

International knee documentation committee (IKDC) score increased almost 26.5 grades postoperatively, which is statistically significant (85 ± 3 Vs. 58.5 ± 5, *P* < 0.001). The mean Lysholm score significantly improved by 27.5 grades at the last follow-up (63.5 ± 3 Vs. 91 ± 2, *P* < 0.001) (Fig. 4).

All patients had returned to their previous activity levels by the final follow-up. One patient suffered from sporadic pain 2 years after the surgery, which was resolved within the following year. No patients reported any recurrence of locking. Moreover, no patients underwent reoperation, and no complications associated with posterior arthroscopy were reported.

## Discussion

This study showed that posterior knee arthroscopy facilitates the safe and effective all-inside repair of PMF tears using a suture hook technique. To the best of our knowledge, this is the first study to evaluate HLM repair using posteromedial transseptal and posterolateral portals.

The loose attachment of the lateral meniscus to the capsule is interrupted by the popliteal hiatus [9, 26]. At the popliteal hiatus, three PMFs (i.e., anteroinferior, posterosuperior, and posteroinferior) [8, 18, 27], along with the popliteus tendon, attach to the lateral meniscus [10]. These PMFs prevent the anterior displacement of the posterolateral corner of the lateral meniscus during knee flexion [28]. Disruption of the posterosuperior PMF is required to induce hypermobility in the lateral meniscus [2]. Meanwhile, the disruption of PMF in MRI is specified as the absence of continued linear structures or water-signal areas between the posterior horn of the lateral meniscus and the joint capsule [29, 30]. However, it is challenging to define PMF tears using MRI [31], meaning that high clinical suspicion is needed to ensure accurate diagnoses.

The complete repair of an abnormal posterosuperior PMF could cause locking symptoms to disappear permanently [8]. Due to improvements in arthroscopic techniques, the recommended treatment for HLM is to preserve the lateral meniscus using various arthroscopic techniques [32, 33]. Inside-out and outside-in techniques have yielded favorable outcomes when the lateral meniscus tear is in the posterior position. However, such techniques are associated with a risk of injury to nerves and blood vessels. At the same time, it is difficult to achieve anatomical reductions or vertical suturing [34–37]. Non-anatomical repair of the lateral meniscus causes excessive stretching and reduced lateral meniscus mobility over time [38].

The all-inside suturing technique provides surgeons with a relatively easy way to suture vertically in posterior horn tears [39, 40]. However, some concerns have arisen regarding complications such as implant irritation, cystic formation, and high costs [26, 36, 37, 41, 42]. In 2006 and 2017, Ahn et al. [31, 43] reported encouraging results associated with the suture hook technique for repairing HLM using the arthroscopic all-inside method through the posterolateral portal. They used 30- and 70-degree lenses sequentially. However, changing the lens during operation can waste time. Also, 70-degree lenses are costly and not available in all operating rooms.

We used only a 30-degree lens to treat cases. The anteromedial portal was used as a viewing portal following Ahn’s technique. We used the posteromedial transseptal and posterolateral portals as viewing and working portals, respectively, making our technique more convenient than Ahn’s technique. The two studies are similar in that both employed the suture hook technique to make the vertical mattress sutures as strong as possible. Like Ahn et al., we could assess the full extent of the lesion and lift the sagging to improve repair accuracy [24].

Steinbacher et al. [13] and Simonetta et al. [44] reported promising clinical results with arthroscopic all-inside lateral meniscus fixation to the posterior capsule using FasT-Fix from anterior portals in the figure-of-four position. In the present study, there is no need to apply the figure-of-four position to elevate the sagging fragment and achieve anatomic reduction. The present technique allowed the surgeon to view the posterolateral compartment easily to anatomically reduce the torn lateral meniscus with the capsule using a vertically oriented suture. Theoretically, this technique allows for strong knot tying while avoiding neurovascular damage and creating a comfortable working position for the surgeon.

The present study has some limitations. For instance, this study lacks biomechanical testing or dynamic MRI for evaluating meniscal excursion after surgery. Another major limitation of this study is the lack of a control group—because of this, the present technique could not be compared with other techniques. Finally, because of the low incidence rate of HLM, the data presented in this study are not sufficient to conclude that the presented technique is the best choice for treating HLM.

## Conclusion

Posterior knee arthroscopy using posteromedial (transseptal) and posterolateral portals facilitates the all-inside repair of disrupted posterosuperior PMF. The favorable clinical results presented in this study show that this repair technique effectively and sufficiently fixes HLM.

## Abbreviations

HLM: Hypermobile lateral meniscus
PMF: Popliteomeniscal Fascicles.
IKDC: International knee documentation committee.
